# Circulating miRNAs as Biomarkers of Tick-Borne Encephalitis Severity: Association with Cytokine Profile in Febrile, Meningeal, and Encephalitic Forms

**DOI:** 10.3390/ijms27146183

**Published:** 2026-07-10

**Authors:** Elena V. Mikheeva, Anna S. Tolmacheva, Mark M. Melamud, Evgeny A. Ermakov, Kseniya S. Aulova, Jialin Li, Yana S. Ulyanova, Elena I. Krasnova, Georgy A. Nevinsky, Anna M. Timofeeva

**Affiliations:** 1 SB RAS Knorre Institute of Chemical Biology and Fundamental Medicine, 630090 Novosibirsk, Russiatolmacheva.anna0301@gmail.com (A.S.T.); evgeny_ermakov@mail.ru (E.A.E.);; 2 Department of Infectious Diseases, Novosibirsk State Medical University, 630091 Novosibirsk, Russia

**Keywords:** tick-borne encephalitis, TBEV, TBEV-Sib, microRNA, miRNA, cytokines, neuroinflammation, biomarkers, stem-loop RT-PCR

## Abstract

Tick-borne encephalitis (TBE) is a neuroinvasive flavivirus infection with a wide spectrum of clinical manifestations whose molecular mechanisms remain insufficiently characterized. MiRNAs regulate pro-inflammatory cytokine production; therefore, changes in their profiles across different forms of TBE may determine the nature of the cytokine response. The aim of this work was to identify the specific features of the circulating miRNA and cytokine profiles in different clinical forms of TBE, as well as to analyze the correlations between them. The study included patients with febrile, meningeal, and encephalitic forms of TBE, patients with inflammatory rheumatic diseases (IRD), and healthy donors. Plasma concentrations of eight miRNAs (miR-25-3p, miR-29a-3p, miR-92a-3p, miR-146a-5p, miR-146b-5p, miR-181a-5p, miR-486-3p, and miR-766-3p) were measured by stem-loop real-time RT-PCR. Cytokine concentrations (IL-1β, IL-2, IL-8, IL-18, TNF-α) were measured by ELISA. Kendall’s rank correlation test was used for the correlation analysis. In all forms of TBE, a distinct circulating miRNA signature emerges (↑ miR-25-3p, miR-146b-5p, ↑ miR-766-3p, and ↓ miR-29a-3p), which is associated with an acute antiviral response and is not specific to chronic autoimmune inflammation. Several miRNAs (miR-29a-3p, miR-92a-3p, miR-146b-5p, and miR-486-3p) showed opposite changes in TBE and IRD, pointing to fundamentally different mechanisms of immune regulation in acute neuroinfection and systemic autoimmune pathology. Several statistical associations between circulating miRNA and pro-inflammatory cytokine levels were identified. TNF-α levels positively correlated with miR-146b-5p and negatively with miR-25-3p, while IL-2 positively correlated with miR-25-3p. In the encephalitic form of TBE, IL-1β positively correlated with miR-146b-5p and miR-92a-3p. The present study is of a pilot nature. The miRNA and cytokine patterns identified here were based on a small sample size and should therefore be regarded as preliminary. Once confirmed, these signatures may provide a basis for the differential diagnosis of TBE and the development of prognostic biomarkers of disease severity.

## 1. Introduction

The etiological agent of tick-borne encephalitis (TBE) is the tick-borne encephalitis virus (TBEV), belonging to the genus *Orthoflavivirus*, family Flaviviridae. The primary vectors and reservoirs of TBEV are the ticks *Ixodes persulcatus* and *Ixodes ricinus* [1]. The geographic range of the virus and the location of its natural foci are closely tied to the distribution of these tick species. For a long time, all TBEV subtypes were grouped within a single paraphyletic species. In February 2026, the European subtype of TBEV (TBEV-Eu) was designated as a distinct species, *Orthoflavivirus neudoerflense*. The remaining TBEV subtypes, including the Siberian subtype (TBEV-Sib), were consolidated into the species *Orthoflavivirus zilberi* [2].

TBEV-Sib circulates in Western Siberia, the Urals, certain regions of Eastern Europe, and Finland [3]. In Western Siberia, TBE incidence has remained high for many years. Tick bites are frequent, and 2.6–5.7% of bitten individuals are hospitalized annually with suspected TBE [4]. TBEV-Sib infection often follows a biphasic course: an initial febrile (influenza-like) phase, followed by more severe neurological involvement, including meningeal and encephalitic forms [5,6].

TBE remains a growing public health concern. Despite the availability of effective vaccines, incidence continues to rise due to the widespread distribution of ticks [7,8]. Ongoing climate change is a key contributing factor, as it increases tick abundance and expands their geographic range [9,10].

Such alarming epidemiological trends highlight the need for a deeper understanding of the molecular basis of TBE pathogenesis. Among the molecular mechanisms involved, miRNAs emerge as key players. These short non-coding RNAs (20–25 nt in length) regulate a wide range of physiological processes, including inflammatory responses and immune reactions during viral infections [11,12].

MiRNAs may also serve as valuable diagnostic and prognostic biomarkers in TBE. Changes in the expression of specific miRNAs have been shown to correlate with disease severity and the risk of complications [13,14]. Given the particularly severe clinical course and high tendency toward chronicity associated with TBEV-Sib, identifying miRNA signatures that predict an unfavorable prognosis is of exceptional clinical importance.

The present study aims to characterize the differential expression of circulating miRNAs and the cytokine profile across different clinical forms of TBE. Specifically, we seek to identify associations that distinguish acute viral inflammation from a systemic autoimmune process, and to define molecular signatures associated with symptom severity and immune dysregulation.

## 2. Results

### 2.1. Demographic and Clinical Characteristics of Patients and Healthy Donors

Five experimental groups were formed. The main group consisted of patients with a verified diagnosis of TBE, divided into three subgroups according to clinical form: Febrile (*n* = 11), Meningeal (*n* = 20), and Encephalitic (*n* = 21). The comparison groups consisted of patients with inflammatory rheumatic diseases (IRD) (*n* = 16) and healthy donors (*n* = 20).

Patients with the febrile form of TBE ranged in age from 21 to 69 years (6 male, 5 female). One patient had incomplete vaccination; the rest were unvaccinated. Fever was low-grade in 55.5% of patients, 38–39 °C in 22.25%, and 39–40 °C in 22.25%. In all patients, the duration of fever did not exceed 5 days. Headache was observed in 100% of patients, nausea in 55.5%, myalgias in 33.3%, and dizziness in 44.4%. Lumbar puncture revealed a cerebrospinal fluid (CSF) cell count of 1 cell/µL in all patients.

Patients with the meningeal form of TBE ranged in age from 18 to 82 years (10 male, 10 female). One patient had been vaccinated in 2009; the remaining patients were unvaccinated. Low-grade fever was observed in two patients, 38–39 °C in eight, and 39–40 °C in ten. Headache was observed in 100% of patients, nausea in 83.3%, vomiting in 27.7%, and myalgias in 44.4%. Two patients developed weakness, numbness in the extremities, and tremor; one patient had an unsteady gait; and one patient experienced transient motor aphasia. The CSF cell count ranged from 14 to 562 cells/µL. Lymphocytic pleocytosis was observed in 72.2% of cases.

Patients with the encephalitic form of TBE ranged in age from 24 to 86 years (12 male, 9 female). None were vaccinated. All patients had a fever of 39–40 °C lasting 3–10 days. On admission, two patients were in stupor (GCS 13–14), one had experienced seizures, one presented with sensorimotor aphasia, and one with tetraparesis. Headache, myalgias, dizziness, unsteady gait, and tremor were observed in 100% of patients. Within 2–5 days, depression of consciousness developed, and patients were transferred to the intensive care unit (ICU). In 33.3% of cases, patients required mechanical ventilation (MV). The CSF cell count ranged from 11 to 857 cells/µL; neutrophilic pleocytosis was observed in 42.8% of cases.

Patients with IRD ranged in age from 21 to 72 years (4 male, 12 female), with confirmed diagnoses of rheumatoid arthritis (RA) (*n* = 5), axial spondyloarthritis (axSpA) (*n* = 5), or systemic lupus erythematosus (SLE) (*n* = 6); most were experiencing a disease flare at the time of enrollment. The control group consisted of apparently healthy volunteers aged 27 to 57 years (10 male, 10 female). The IRD group was intentionally included as a heterogeneous autoimmune comparator to distinguish miRNA changes specific to acute viral infection from those associated with chronic systemic inflammation broadly. However, the distinct immunopathological mechanisms of RA, axSpA, and SLE may differentially influence miRNA and cytokine profiles, and this heterogeneity is acknowledged as a limitation (see Section 3.4).

The sex distribution did not differ significantly across the five groups (χ^2^ = 4.35, df = 4, *p* = 0.36). The IRD group had a higher proportion of women (75.0%), consistent with the known female predominance in RA and SLE [15,16]; however, this difference was not statistically significant compared to the control group (Fisher’s exact test, *p* = 0.18). The control group had a narrower age range compared to the TBE subgroups and the IRD group, which should be considered as a potential confounder (see Section 3.4).

Blood samples were collected at hospital admission. The median time from symptom onset to blood collection was 3 days (range 2–9) in the febrile group, 5 days (range 2–>10) in the meningeal group, and 5 days (range 1–>5) in the encephalitic group. In the meningeal group, 5 of 20 patients were admitted after day 10; in the encephalitic group, 8 of 21 patients were admitted after day 5.

### 2.2. Profile of Circulating miRNAs in Various Clinical Forms of Tick-Borne Encephalitis and in Comparison Groups

A panel of eight miRNAs (miR-25-3p, miR-29a-3p, miR-92a-3p, miR-146a-5p, miR-146b-5p, miR-181a-5p, miR-486-3p, and miR-766-3p) was selected for quantitative analysis based on their documented roles in innate immune regulation, adaptive immunity, interferon response, and neuroinflammation [11,17,18]. MiRNA quantification was performed using the stem–loop reverse transcription and real-time PCR (SL RT-PCR) method, which provides high specificity in distinguishing miRNAs with closely related sequences.

**Figure 1:**
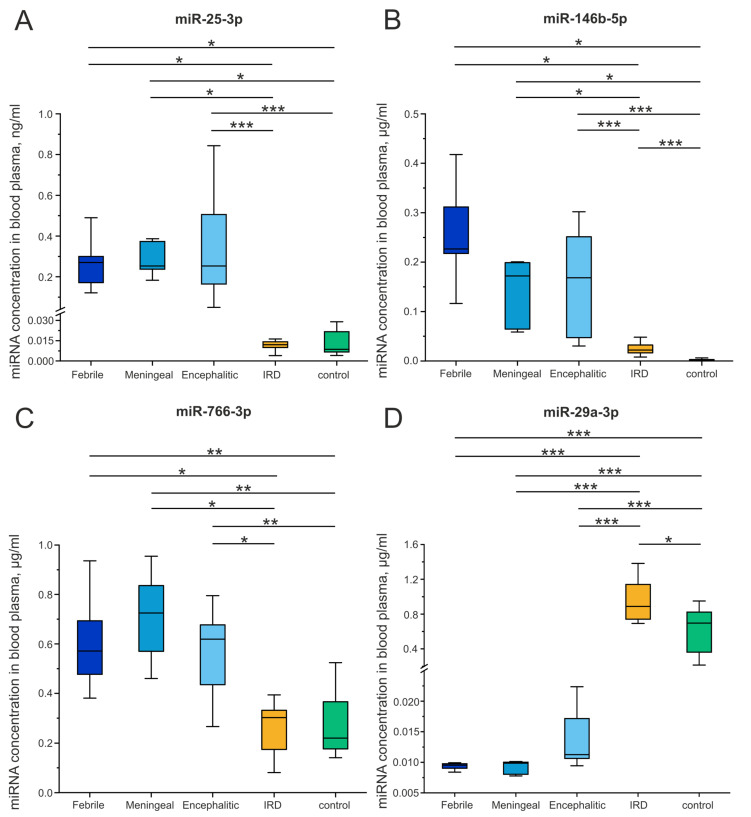
Analysis of miRNA concentrations ((**A**): miR-25-3p, (**B**): miR-146b-5p, (**C**): miR-766-3p, (**D**): miR-29a-3p) in blood plasma from patients with febrile, meningeal, and encephalitic forms of tick-borne encephalitis (TBE), patients with inflammatory rheumatic diseases (IRD), and healthy individuals (control). The Kruskal–Wallis test was used to analyze differences, and the Mann–Whitney test for pairwise comparisons. Significance levels: * *p* < 0.05, ** *p* < 0.01, *** *p* < 0.001.

Significantly elevated levels of miR-25-3p, miR-146b-5p, and miR-766-3p were found in all TBE patient groups relative to both healthy donors and patients with IRD (Figure 1A–C). In contrast, miR-29a-3p levels were decreased in all TBE patient groups compared to both comparison groups (Figure 1D). Elevated levels of miR-146b-5p and miR-29a-3p were also observed in patients with IRD relative to the control group. These findings suggest that miR-25-3p, miR-29a-3p, and miR-766-3p are associated with an acute antiviral response during TBEV infection, rather than with the chronic inflammatory process typical of autoimmune pathology.

**Figure 2:**
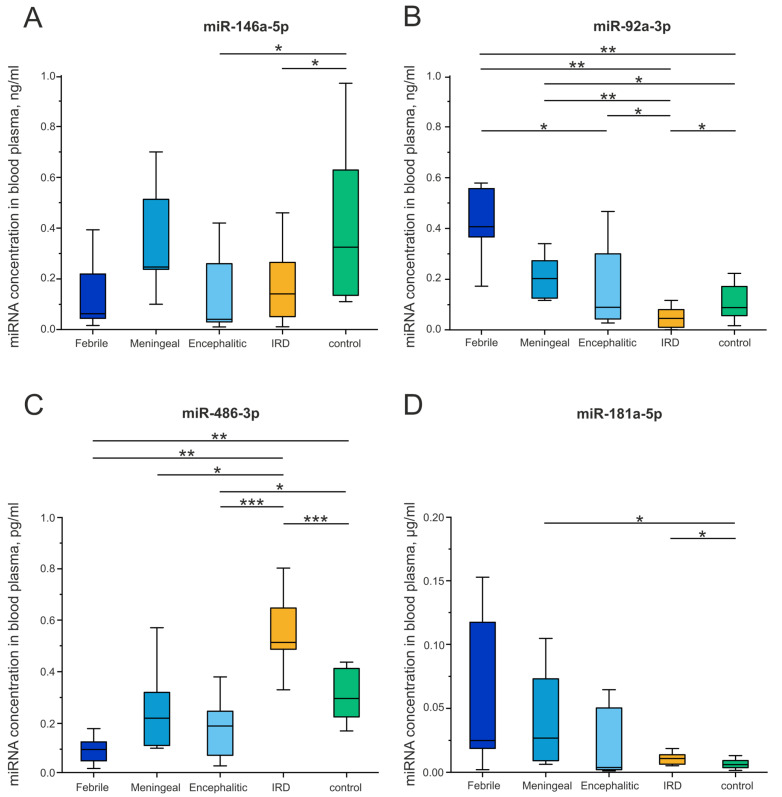
Analysis of miRNA concentrations ((**A**): miR-146a-5p, (**B**): miR-92a-3p, (**C**): miR-486-3p, (**D**): miR-181a-5p) in blood plasma from patients with febrile, meningeal, and encephalitic forms of TBE, patients with IRD, and healthy individuals (control). The Kruskal–Wallis test was used to analyze differences, and the Mann–Whitney test for pairwise comparisons. Significance levels: *p < 0.05, ** *p* < 0.01, *** *p* < 0.001.

A decrease in miR-146a-5p levels was observed in patients with the encephalitic form of TBE and in the IRD group relative to healthy controls (Figure 2A). miR-146a-5p acts as one of the key factors limiting excessive activation of immune responses [19]. In the encephalitic form of TBE, virus-mediated damage of nervous tissue triggers an excessive neuroinflammatory cascade. The observed decrease in miR-146a-5p under these conditions may reflect depletion of this regulatory molecule. Reduced expression of miR-146a has been documented in a wide range of systemic autoimmune and autoinflammatory diseases [19,20]. Deficiency of miR-146a-5p is considered a factor that contributes to the maintenance of chronic inflammation, which is consistent with the low miR-146a-5p levels observed in the IRD group.

The concentration of miR-92a-3p was elevated in patients with febrile and meningeal forms of TBE compared to the IRD group and healthy donors. In the IRD group, miR-92a-3p levels were decreased relative to healthy donors (*p* = 0.03) (Figure 2B). miR-92a-3p is involved in the regulation of inflammation, apoptosis, and endothelial integrity [21,22]. The elevation of miR-92a-3p in plasma in febrile and meningeal forms of TBE, which are characterized by peak viremia, likely reflects activation and damage of endothelial cells in response to TBEV infection. The absence of a significant increase in miR-92a-3p in patients with the encephalitic form of TBE is consistent with viral redistribution from the systemic circulation into the CNS, where neuroinflammation predominates over systemic endothelial activation. In the IRD group, miR-92a-3p levels were decreased relative to healthy donors, consistent with reported suppression of this miRNA in chronic autoimmune conditions [22]. This creates an opposite pattern to that seen in acute TBE infection, which may offer differential diagnostic value.

Levels of miR-486-3p were decreased in patients with febrile (*p* = 0.007) and encephalitic (*p* = 0.02) forms of TBE compared to healthy donors (Figure 2C). The main sources of circulating miR-486-3p are erythrocytes and platelets [23,24]. This decrease likely reflects virus-induced suppression of erythropoiesis and platelet activation under acute inflammatory conditions [25,26]. In contrast, elevated miR-486-3p in IRD reflects chronic activation of erythroid precursors [27,28]. Thus, miR-486-3p shows opposite patterns in acute TBE infection and chronic autoimmune inflammation, highlighting its potential differential diagnostic value.

Levels of miR-181a-5p were elevated in patients with the meningeal form of TBE and in the IRD group compared to healthy donors (Figure 2D). The elevation of miR-181a-5p in IRD is consistent with literature data on its overexpression in systemic autoimmune diseases [29].

### 2.3. Cytokine Profile in Various Clinical Forms of Tick-Borne Encephalitis and in Comparison Groups

Changes in circulating miRNA concentrations in TBE patients may be relevant to the regulation of the inflammatory response, including pro-inflammatory cytokine production. Therefore, plasma concentrations of key pro-inflammatory cytokines (IL-1β, IL-2, IL-8, IL-18, and TNF-α) were analyzed in patients with different clinical forms of TBE (febrile, meningeal, and encephalitic), as well as in healthy donors and patients with IRD.

**Figure 3:**
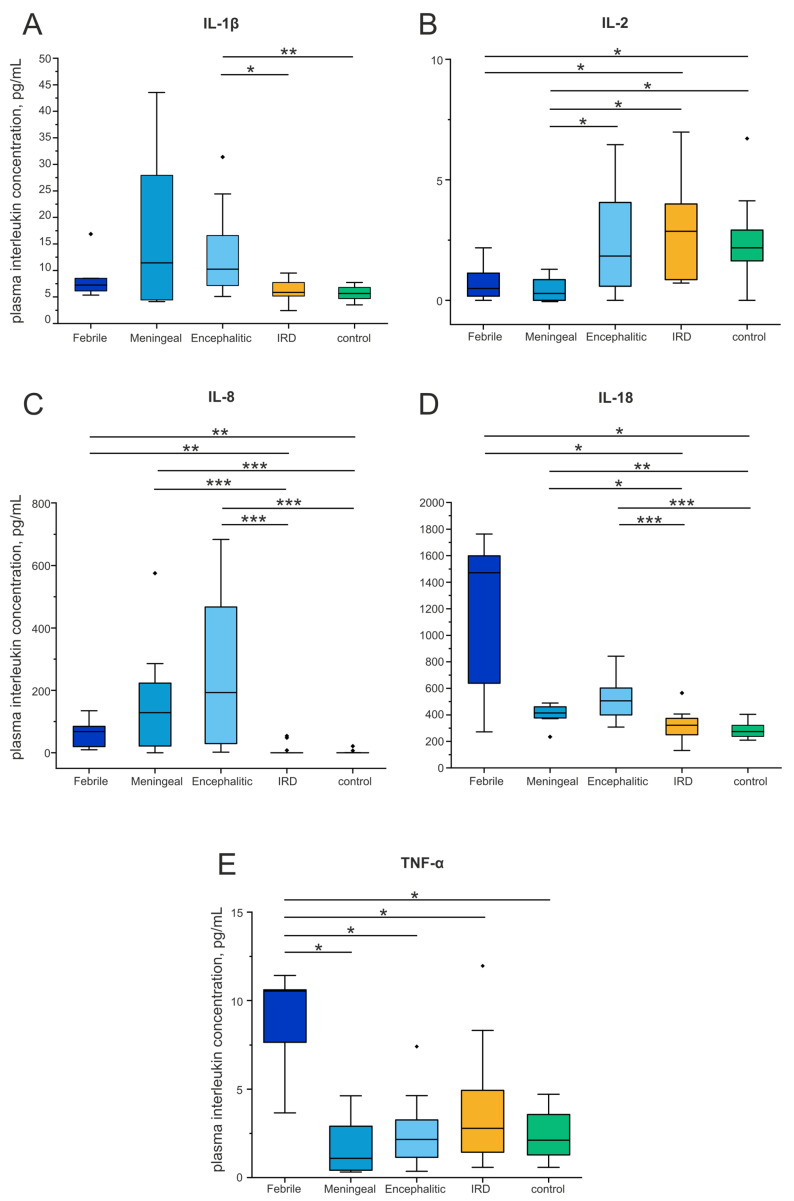
Analysis of the levels of key pro-inflammatory cytokines: IL-1β (**A**), IL-2 (**B**), IL-8 (**C**), IL-18 (**D**), and TNF-α (**E**) in the blood plasma of patients with febrile, meningeal, and encephalitic forms of TBE, patients with IRD, and healthy individuals. The Kruskal–Wallis test was used to analyze differences, and the Mann–Whitney test was employed for pairwise comparisons. Significance levels: *p < 0.05, ** *p* < 0.01, *** *p* < 0.001.

IL-1β concentration was significantly higher in patients with the encephalitic form of TBE than in healthy donors (*p* < 0.001) and IRD patients (*p* = 0.002) (Figure 3A). IL-1β is a key pro-inflammatory cytokine that plays an important role in the pathogenesis of various infections. Its elevated concentration in the encephalitic form of TBE is consistent with its role as a mediator of neuroinflammation in severe disease with CNS involvement [30,31]. The trend toward increased IL-1β in the meningeal form, which did not reach statistical significance, may reflect less pronounced involvement of the brain parenchyma compared to the encephalitic form.

In patients with the meningeal form of TBE, plasma IL-2 concentration was significantly lower than in the IRD group (*p* = 0.002) and healthy donors (*p* = 0.001) (Figure 3B). A similar decrease in IL-2 levels was observed in the febrile form of TBE compared to IRD (*p* = 0.018) and healthy donors (*p* = 0.038). This decrease in plasma IL-2 in patients with febrile and meningeal forms of TBE may be due to several interrelated mechanisms. First, IL-2 is actively consumed by regulatory T cells (Tregs), leading to a reduction in its circulating concentration during massive immune cell activation in the acute phase of infection [32,33]. Second, high viral load causes functional T-cell exhaustion, resulting in early loss of IL-2 production capacity [34].

Elevated IL-8 concentrations were observed in all forms of TBE compared to healthy donors and IRD patients (Figure 3C). IL-8 concentrations increased in the following order across clinical forms: febrile < meningeal < encephalitic. Previous studies have shown that IL-8 levels are elevated in the serum [35] and CSF [36] of TBE patients. Although IL-8 is not a highly specific predictor of disease severity [37], this stepwise increase is consistent with an intensification of the neuroinflammatory response as the disease becomes more severe.

In all forms of TBE, IL-18 concentrations were significantly higher than in the IRD group and healthy donors (Figure 3D). The highest median IL-18 concentration was observed in the febrile form of TBE (1471.4 [638.9; 1599.4] pg/mL). A similar increase in IL-18 in serum and CSF has been described in encephalitis caused by Japanese encephalitis virus [38], suggesting that this mechanism may be common to neuroinvasive flavivirus infections.

The highest TNF-α concentration (10.53 [7.64; 10.6] pg/mL) was observed in the febrile form of TBE (Figure 3E). TNF-α levels in this group were significantly higher than in the meningeal form (*p* = 0.010), the encephalitic form (*p* = 0.010), and healthy donors (*p* = 0.004). TNF-α is a pleiotropic cytokine with a dual role in flavivirus infections. On one hand, TNF-α plays a protective role by limiting excessive neuroinflammation [39,40]. On the other hand, excessive TNF-α production is associated with blood–brain barrier disruption and neurotoxicity [41]. The peak TNF-α concentrations observed in the febrile form are consistent with experimental data showing that systemic TNF-α reaches maximum levels during the acute phase of infection, when peripheral viremia is highest, representing an adaptive immunoregulatory response aimed at limiting CNS immunopathology [40].

Taken together, these results demonstrate that TBE is associated with a distinct pattern of pro-inflammatory cytokine changes in blood plasma, reflecting the character and intensity of the immune response across different clinical forms.

### 2.4. Associations Between Cytokine Concentrations and Circulating miRNA Levels

To identify possible relationships between the cytokine profile and miRNA levels, a correlation analysis was performed using Kendall’s rank correlation coefficient (τ).

**Figure 4:**
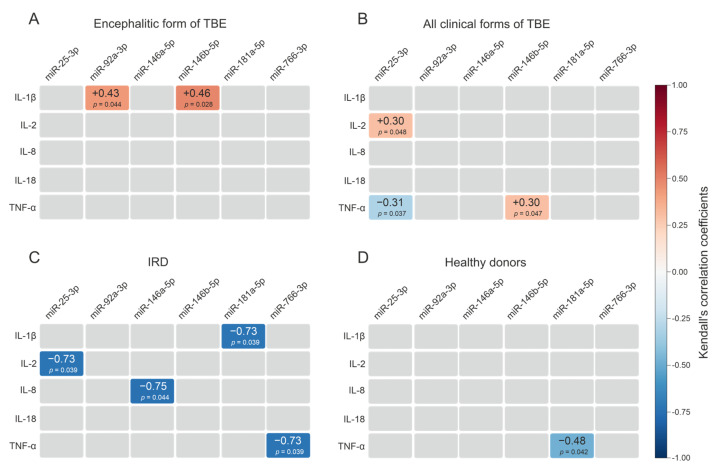
Correlations between circulating miRNA concentrations and pro-inflammatory cytokine concentrations in blood plasma of patients in the study groups ((**A**): encephalitic form of TBE, (**B**): all clinical forms of TBE, (**C**) IRD, (**D**) healthy donors). Color coding reflects Kendall’s correlation coefficients.

In patients with the encephalitic form of TBE, significant positive correlations were identified between IL-1β concentration and the levels of miR-92a-3p (τ = 0.43; *p* = 0.044) and miR-146b-5p (τ = 0.46; *p* = 0.028) (Figure 4A).

In the overall TBE cohort (all clinical forms), TNF-α was positively associated with miR-146b-5p (τ = +0.30; *p* = 0.047) and negatively with miR-25-3p (τ = −0.31; *p* = 0.037). A positive association was also observed between IL-2 and miR-25-3p (τ = +0.30; *p* = 0.048) (Figure 4B).

In the IRD group, significant negative associations were identified between pro-inflammatory cytokine levels and the expression of several miRNAs: TNF-α was inversely associated with miR-766-3p (τ = −0.73; *p* = 0.039), IL-1β with miR-181a-5p (τ = −0.73; *p* = 0.039), and IL-8 with miR-146a-5p (τ = −0.75; *p* = 0.044). In addition, IL-2 and miR-25-3p were negatively associated (τ = −0.73; *p* = 0.039) (Figure 4C).

In the healthy donor group, a negative association was found between TNF-α concentration and miR-181a-5p levels (τ = −0.48; *p* = 0.042) (Figure 4D).

## 3. Discussion

TBE is a neuroinvasive flavivirus infection characterized by a wide spectrum of clinical manifestations, ranging from the relatively benign febrile form to severe encephalitic CNS damage [42]. Despite progress in studying TBE pathogenesis, the molecular mechanisms determining disease severity remain poorly characterized.

The present study compared circulating miRNA profiles in patients with three clinical forms of TBE against those in patients with IRD and healthy donors. The IRD group was included to distinguish miRNA profile changes specific to viral neuroinfection from the immunoinflammatory patterns characteristic of autoimmune pathology.

One of the key mechanisms linking viral infections and autoimmune diseases is molecular mimicry. Similarity in amino acid sequences between a viral antigen and a host autoantigen allows pathogens to evade immune defense, gaining an evolutionary advantage [43]. This similarity can lead to activation of autoreactive T and B cells that cross-recognize self-antigens. Molecular mimicry has been described for a wide range of viruses, including Epstein–Barr virus, herpes simplex virus, Coxsackie viruses, and SARS-CoV-2 [44,45,46,47].

Flaviviruses, including TBEV, exhibit pronounced neurotropism and can induce a robust inflammatory response in the CNS [48], potentially creating conditions for autoimmune activation. In this context, comparing miRNA profiles in TBE and IRD is of particular interest for identifying shared and disease-specific molecular patterns of immune regulation.

### 3.1. A Common miRNA Signature in TBE: Markers of Acute Antiviral Response

The results reveal several functionally significant patterns reflecting both common mechanisms of the antiviral response and specific changes associated with disease severity.

The miRNA signature identified across all forms of TBE (↑ miR-25-3p, ↑ miR-766-3p, ↓ miR-29a-3p) may reflects a balance between antiviral defense and the limitation of excessive inflammation, suggesting that these miRNAs could potentially serve as universal markers of the acute antiviral response in TBE.

In addition, several miRNA changes were identified that are typical of individual TBE forms and may help distinguish acute viral neuroinflammation from chronic autoimmune pathology. The change in miR-92a-3p (↑ in febrile and meningeal forms of TBE; ↓ in IRD) may reflect endothelial activation in acute viral infection and its suppression in chronic inflammation. The divergent dynamics of miR-486-3p (in febrile and encephalitic forms of TBE; ↑ in IRD) may reflect opposing shifts in the erythroid and platelet compartments, with suppression during acute viral infection and possible activation during chronic inflammation.

### 3.2. Cytokine Profile in TBE and Its Relationship with miRNA Profiles

A distinct pattern of pro-inflammatory cytokine changes was identified in TBE patients, reflecting the character and intensity of the immune response across the different clinical forms. IL-1β concentrations was significantly elevated in the encephalitic form of TBE, consistent with the key role of this cytokine in neuroinflammation. IL-8 and IL-18 levels were elevated in all three forms of TBE, with IL-8 concentrations increasing in the following order: febrile < meningeal < encephalitic, consistent with progressive intensification of the inflammatory response. Previous studies have shown that infection of microglial cells and astrocytes with Japanese encephalitis virus induces IL-18 production, leading to activation of these cells and reduced neuronal survival [38].

Elevated miR-25-3p suppresses the TLR4/NLRP3 axis, reducing inflammasome activation and constraining IL-1β and IL-18 production [49]. Mechanistically, miR-25-3p targets *TLR4* directly, thereby attenuating downstream MyD88-dependent NF-κB signaling and limiting NLRP3 inflammasome assembly and caspase-1-mediated cytokine maturation. Concurrently, elevated miR-766-3p contributes to anti-inflammatory regulation through indirect inhibition of NF-κB signaling [50], and has been shown to reduce neuroinflammation in CNS injury models [51], suggesting a potential neuroprotective role in the context of TBEV-associated neuroinflammation.

Alternatively, decreased miR-29a-3p is associated with upregulation of TNF-α and IL-1β. miR-29a-3p targets *TNFRSF1A* (the gene encoding TNF receptor 1) thereby suppressing NF-κB-dependent pro-inflammatory cytokine production [52,53]. Downregulation of miR-29a-3p during TBE thus releases this brake, potentially permitting upregulation of TNF-α and IL-1β through enhanced TNFRSF1A/NF-κB signaling [54].

The opposing regulatory effects of these miRNAs (miR-25-3p and miR-766-3p suppressing, and miR-29a-3p downregulation promoting, pro-inflammatory cytokine production) may partially cancel each other out, providing a plausible mechanistic explanation for the absence of significant differences in IL-1β and IL-18 levels observed across TBE patient groups.

The increase in miR-146b-5p in TBE is consistent with a role in limiting NF-κB-dependent production of pro-inflammatory cytokines [55]. Mechanistically, miR-146b-5p directly targets *TRAF6*, *IRAK1*, and *RELA*, which are the key upstream activators of the NF-κB signaling cascade, thereby attenuating TLR- and IL-1R-mediated inflammatory signaling [56,57]. Overexpression of miR-146b-5p has been shown to reduce pro-inflammatory cytokine production in CNS injury models through inhibition of the IRAK1/TRAF6/TAK1/NF-κB axis [58], raising the speculative possibility that elevated circulating miR-146b-5p in TBE may reflect an attempt to limit neuroinflammatory damage, though whether circulating levels reflect CNS-derived miRNA or peripheral immune cell activity remains unclear.

The decrease in miR-146a-5p in the encephalitic form may contribute to intensified neuroinflammation through disinhibition of NF-κB signaling in glial cells [59]. Mechanistically, miR-146a-5p functions as a negative-feedback regulator of innate immune signaling by targeting *IRAK1* and *TRAF6*, thereby suppressing NF-κB-driven production of TNF-α, IL-1β and IL-6 in microglia and astrocytes [60,61,62]. Loss of this regulatory restraint in the encephalitic form may permit sustained NF-κB activation in glial cells, amplifying neuroinflammatory cascades and potentially contributing to blood–brain barrier disruption and neuronal damage.

It should be emphasized that all mechanistic interpretations presented above are speculative and are offered solely as hypothesis-generating context. The described molecular mechanisms have been established in other biological systems and disease contexts, as cited, but have not been directly demonstrated in the context of TBEV infection or in the patient cohort described here.

Correlation analysis identified several statistical relationships between cytokines and miRNAs whose patterns differed across study groups and may reflect group-specific features of the immune response. In the encephalitic form of TBE, the association between IL-1β and miR-146b-5p is consistent with a known regulatory mechanism. IL-1β activates the transcription factor NF-κB, which induces the transcription of miR-146b-5p [63]. In turn, miR-146b-5p suppresses the adaptor proteins TRAF6 and IRAK1, thereby limiting further NF-κB activation [56]. This forms a negative feedback loop, in which an increase in the pro-inflammatory signal (↑ IL-1β) triggers induction of a limiting mechanism (↑ miR-146b-5p). miR-92a-3p is involved in the regulation of apoptosis [64]. The correlation between IL-1β and miR-92a-3p levels may reflect compensatory activation of anti-apoptotic mechanisms under conditions of severe neuroinflammation characteristic of the encephalitic form.

In the TBE cohort, the positive correlations between TNF-α and miR-146b-5p (τ = +0.30; *p* = 0.047) is consistent with a similar negative feedback mechanism, since TNF-α also activates NF-κB [65,66]. The inverse association between TNF-α and miR-25-3p (τ = −0.31; *p* = 0.037) is in line with published evidence that TNF-α can activate pro-apoptotic cascades [67,68], while miR-25-3p possesses anti-apoptotic activity [69,70].

In the IRD group, the negative correlations between IL-8 and miR-146a-5p (τ = −0.75; *p* = 0.044) is consistent with the reported deficiency of miR-146a-5p in autoimmune diseases [71,72]. The opposing directions of the IL-2–miR-25-3p relationship in viral (positive in TBE) and autoimmune (negative in IRD) inflammation suggest that the same regulatory axis may operate in two substantially different modes; however, this interpretation should be treated with caution given the small and heterogeneous IRD group (comprising patients with RA, axSpA and SLE).

In the healthy donor group, the negative association between TNF-α and miR-181a-5p (τ = −0.48; *p* = 0.042) is consistent with published evidence that TNF-α can modulate miR-181a expression [73,74]. Nevertheless, a causal interpretation cannot be supported by correlational data, and the specific mechanism in healthy individuals remains poorly understood.

It should be emphasized that all relationships reported above are statistical associations derived from rank correlation analysis in relatively small and clinically heterogeneous groups, and they do not establish directionality, temporal sequence, or causality between cytokines and circulating miRNAs. Moreover, correlation observed in plasma samples cannot discriminate between scenarios in which miRNA modulates cytokine production. The opposite directions of the IL-2–miR-25-3p association in the TBE and IRD groups illustrate how context-dependent and non-deterministic such relationships can be. In addition, plasma miRNA levels reflect contributions from multiple cellular sources (immune cells, endothelium, extracellular vesicles), so a peripheral correlation does not necessarily mirror events at the tissue level. The net effect of simultaneous changes in multiple miRNAs on cytokine output is difficult to predict without direct experimental evidence, and the observed cytokine levels likely reflect the combined influence of many regulatory factors beyond the miRNAs measured here.

The mechanistic interpretations proposed above (the IL-1β/TNF-α → NF-κB → miR-146b-5p negative feedback loop, the antiapoptotic role of miR-25-3p under TNF-α-driven inflammation, and the modulation of miR-181a by TNF-α) are consistent with previously published functional and regulatory data, but in the present study they remain hypothetical working models. Their validation will require larger, prospectively recruited and clinically more homogeneous cohorts, longitudinal sampling to resolve the temporal order of cytokine and miRNA changes, and dedicated in vitro and in vivo experiments to formally test causality and directionality.

### 3.3. Matching with Signatures in Autoimmune Diseases

When interpreting the IRD comparisons, it is important to note that the IRD group comprised three distinct autoimmune conditions (RA, axSpA, SLE) with different immunopathological mechanisms. The following comparisons therefore reflect general patterns of autoimmune inflammation rather than disease-specific signatures.

Increased miR-146a-5p [72] and miR-92a-3p [75], along with changes in other miRNAs involved in TLR signaling and B-cell activation, have been described in SLE [76]. In rheumatoid arthritis, elevated miR-146a levels and changes in the miR-29a profile have been reported [77,78]. In dermatomyositis, increases in miR-146a-5p and miR-146b-5p have been reported [79]. A meta-analysis of differentially expressed miRNAs in autoimmune diseases identified miR-146a as one of the most consistently altered miRNAs across various autoimmune pathologies [80]. The miRNA pattern in TBE partially overlaps with the autoimmune pattern: miR-146b-5p is elevated in both TBE and several autoimmune diseases, whereas decreased miR-146a-5p in the encephalitic form of TBE may represent a feature that could help differentiate severe neuroinfection from autoimmune pathology.

The decrease in miR-25-3p in IRD patients is consistent with diminished miR-25-3p levels reported in rheumatoid arthritis [81] and osteoarthritis [82]. The opposing directions of the IL-2–miR-25-3p correlation in IRD (τ = −0.73) and TBE (τ = +0.30) are noteworthy and may suggest different regulatory modes of these molecules in viral versus autoimmune inflammation.

SLE is characterized by marked cytokine dysregulation, including elevated IL-1β, IL-6, IL-10, TNF-α, and several chemokines [83], driven by systemic inflammation that is fundamentally distinct from that seen in acute viral infection.

### 3.4. Limitations

The present work should be regarded as an exploratory, pilot study, and its findings as preliminary and hypothesis-generating rather than confirmatory. Several limitations must be taken into account when interpreting the data.

First, the sample size is relatively small. The limited sample size reduces statistical power. These findings require validation in larger, independently recruited cohorts.

Second, the age and sex distributions across groups were not formally matched. The control group (age 27–57 years) was younger on average than the meningeal (18–82) and encephalitic (24–86) TBE subgroups. Both age and sex are known to influence circulating miRNA profiles, and the potential confounding effect of these variables cannot be entirely excluded. Future studies should employ age- and sex-matched designs or include these variables as covariates in multivariate models.

Third, the miRNA analysis was limited to a targeted panel of eight miRNAs selected on the basis of their documented roles in immune regulation and neuroinflammation. Similarly, the cytokine analysis was restricted to five pro-inflammatory cytokines, and a broader panel including anti-inflammatory and regulatory cytokines would provide a more complete picture of the immune response.

Fourth, the IRD comparison group was heterogeneous, comprising patients with RA (*n* = 5), axSpA (*n* = 5), and SLE (*n* = 6). While all three conditions share a common foundation of chronic immune dysregulation and loss of immune self-tolerance [84,85], their dominant immunopathological mechanisms differ substantially. RA is characterized by synovial hyperplasia, autoantibody production, and TNF-α/IL-6-driven joint destruction [86]. axSpA is driven predominantly by the IL-23/IL-17 axis, with entheseal inflammation and new bone formation as hallmarks [87,88]. SLE is distinguished by immune complex deposition and systemic multi-organ involvement [89,90]. These mechanistic differences may translate into distinct miRNA and cytokine profiles, such that pooling the three conditions into a single comparison group may increase within-group variability, reduce the specificity of observed differences relative to TBE, and potentially obscure condition-specific patterns. With subgroup sizes of *n* = 5–6, formal statistical subgroup analysis would be severely underpowered and was therefore not performed. Conclusions drawn from comparisons involving the IRD group should accordingly be interpreted with caution, and disease-specific interpretation of IRD-associated findings requires replication in larger, diagnosis-stratified cohorts.

Fifth, it is important to note that the reported correlations identify statistical associations and do not establish causality or directionality. The mechanistic interpretations proposed are based on consistency with published functional studies and should be considered hypothetical until validated experimentally.

## 4. Materials and Methods

### 4.1. Donors and Patients

This study was approved by the Local Ethics Committee of the Institute of Chemical Biology and Fundamental Medicine (ICBFM) SB RAS (protocol No. 26-7 of 24 April 2025). All participants (patients and healthy donors) were informed about the purposes of the study and gave written informed consent to blood collection in accordance with the principles of the Declaration of Helsinki.

Patients diagnosed with TBE were treated at City Infectious Clinical Hospital No. 1 in Novosibirsk. Blood samples were collected during the acute phase of the disease. Patients were eligible for inclusion if they had no chronic coinfections or autoimmune diseases.

Two groups were formed as comparison groups: patients with inflammatory rheumatic diseases (IRD) and healthy donors. The IRD group included patients with a confirmed diagnosis of RA, axSpA, or SLE.

Venous blood was collected in the fasting state using vacuum tubes with clotting activators. Blood samples were centrifuged at 3000× *g* for 10 min. Plasma samples were frozen and stored at −70 °C until analysis. Anti-TBEV antibody titers were determined using the D-1156 kit (Vector-Best, Novosibirsk, Russia) according to the manufacturer’s instructions.

### 4.2. Quantification of miRNA by Stem-Loop RT-qPCR

A miRNA isolation kit (catalog No. LRU-100-50-N, Biolabmix, Novosibirsk, Russia) was used to isolate miRNA from 200 µL of blood plasma according to the manufacturer’s instructions.

The analysis was performed in two stages. At the reverse transcription stage, a stem-loop (SL) primer specifically binds to the target miRNA and initiates cDNA synthesis. At the real-time PCR stage, the resulting cDNA is amplified using a forward primer complementary to the miRNA sequence and a universal reverse primer [91,92].

Reverse transcription was performed using a commercial RT M-MuLV-RH kit (catalog No. R03-10, Biolabmix, Novosibirsk, Russia) in combination with a specific stem-loop (SL) primer for each miRNA (Table 1), as described in [92]. The reaction mixture consisted of 2 µL of SL-primer (1 µM), 2 µL of total isolated miRNA, and 8 µL of diethyl pyrocarbonate (DEPC)-treated water. This mixture was incubated at 70 °C for 3 min, after which the samples were immediately transferred to an ice bath. Subsequently, 4 µL of 5× RT buffer, 1 µL of M-MuLV-RH reverse transcriptase (10 U/µL), and 3 µL of DEPC-treated water were added to the mixture.

Reverse transcription was performed according to the following protocol: 30 min at 16 °C; (30 s at 30 °C, 30 s at 42 °C, 1 s at 50 °C) × 45 cycles; 5 min at 85 °C.

The resulting cDNA was analyzed using the BioMaster HS-qPCR PCR kit (Biolabmix, Novosibirsk, Russia). The reaction mixture (20 µL) contained 10 µL of BioMaster HS-qPCR (2×), 0.4 µL of forward primer (10 µM) (Table 1), 0.4 µL of universal reverse primer (5′-GTGCAGGGTCCGAGGT-3′), 0.2 µL of universal probe (10 µM) ([FAM]CAGAGGAAGTGCAAGGTC[BHQ1]), 2 µL of cDNA (SL-RT product), and 7 µL of DEPC-treated water.

The amplification procedure included the following steps: 5 min at 95 °C, (20 s at 95 °C and 1 min at 55 °C) × 45 cycles. Fluorescence of the FAM signal was measured at the end of each cycle.

Quantification of miRNA in blood plasma was performed using absolute quantification. For each miRNA, a calibration curve was constructed using ten-fold serial dilutions of the corresponding synthetic oligonucleotide (with a sequence identical to the mature miRNA) over a concentration range of 10^−3^ to 10^−9^ ng/μL.

### 4.3. Cytokine Quantification by ELISA

Plasma concentrations of IL-1β, IL-2, IL-8, IL-18, and TNF-α were measured by ELISA using commercial kits (catalog Nos. A-8756, A-8772, A-8762, A-8770, and A-8766; Vector-Best, Novosibirsk, Russia), according to the manufacturer’s instructions.

### 4.4. Statistical Analysis

Statistical data processing was performed using STATISTICA 10 software (StatSoft, Tulsa, OK, USA). Data visualization was carried out using OriginPro 2021 (OriginLab, Northampton, MA, USA). Most variables had a non-normal distribution (Shapiro–Wilk test); therefore, non-parametric tests were used to analyze the significance of differences. The significance of differences among several groups was calculated using the Kruskal–Wallis test. For pairwise comparisons, the Mann–Whitney U test was used. A *p*-value < 0.05 was considered significant.

Kendall’s rank correlation test was used for correlation analysis.

## 5. Conclusions

This study provides the first comprehensive comparative analysis of circulating miRNA and pro-inflammatory cytokine profiles across three clinical forms of TBE (febrile, meningeal, and encephalitic), compared with patients with IRD and healthy donors. The data provide insight into the molecular mechanisms underlying the varying severity of TBE and highlight fundamental differences between acute viral neuroinflammation and chronic autoimmune pathology.

A universal feature of all TBE forms is a specific miRNA signature: elevated miR-25-3p and miR-766-3p, and reduced miR-29a-3p. This signature may reflect an acute antiviral response and may represent candidate molecular marker of TBEV infection. Additional miRNA changes are associated with specific clinical forms: decreased miR-146a-5p is characteristic of the encephalitic form. Divergent changes in miR-92a-3p (elevated in TBE, decreased in IRD) and miR-486-3p (decreased in TBE, elevated in IRD) allow differentiation of acute viral inflammation from chronic autoimmune processes.

Correlation analysis between cytokine and miRNA levels revealed key regulatory relationships. Positive correlations were found between IL-1β/TNF-α and miR-146b-5p, and between IL-1β and miR-92a-3p. The opposing direction of the IL-2–miR-25-3p relationship in TBE (positive) and IRD (negative) suggest that a single regulatory axis can operate in two substantially different modes: adaptive in acute viral infection and maladaptive in chronic autoimmune inflammation.

Taken together, these findings form a coherent picture of the molecular events determining TBE severity. The identified miRNA and cytokine signatures not only deepen our understanding of disease pathogenesis, but also provide a preliminary basis for hypothesis generation regarding novel diagnostic and prognostic biomarkers that could potentially enable early differentiation of acute flavivirus neuroinflammation from systemic autoimmune pathology and prediction of severe encephalitic forms.

## Figures and Tables

**Table 1:** Sequences of SL- and forward primers.

miRNA	SL-Primers	Forward Primers
miR-25-3p	5′-GTTGGCTCTGGTGCAGGGTCCGAGGTATTCGCACCAGAGCCAACTCAGAC-3′	5′-GTTTGCATTGCACTTGTCTCG-3
miR-29a-3p	5′-GTTGGCTCTGGTGCAGGGTCCGAGGTATTCGCACCAGAGCCAACTAACCG-3′	5′-GTTTGGTAGCACCATCTGAAAT-3′
miR-30a-5p	5′-GTTGGCTCTGGTGCAGGGTCCGAGGTATTCGCACCAGAGCCAACCTTCCA-3′	5′-GGGTGTAAACATCCTCGAC-3′
miR-92a-3p	5′-GTTGGCTCTGGTGCAGGGTCCGAGGTATTCGCACCAGAGCCAACACAGGC-3′	5′-GTTTGTATTGCACTTGTCCCG-3′
miR-146a-5p	5′-GTTGGCTCTGGTGCAGGGTCCGAGGTATTCGCACCAGAGCCAACAACCCA-3′	5′-GGTGGTGAGAACTGAATTCCA-3′
miR-146b-5p	5′-GTTGGCTCTGGTGCGGGTCCGAGGTATTGCACCAAGAGCCAACCAGCCT-3′	5′-GTTTTTCGTGAGAACTGAATTCCAT-3′
miR-181a-5p	5′-GTTGGCTCTGGTGCAGGGTCCGAGGTATTCGCACCAGAGCCAAC-3′	5′-GGAACATTCAACGCTGTCG-3′
miR-486-3p	5′-GTTGGCTCTGGTGCAGGGTCCGAGGTATTCGCACCAGAGCCAACCTCGGG-3′	5′-GTTTCCTGTACTGAGCTGC-3′
miR-766-3p	5′-GTTGGCTCTGGTGCAGGGTCCGAGGTATTCGCACCAGAGCCAACGCTGAG-3′	5′-GAGCUUGGGAUAGAGGGCUUA-3′

## Data Availability

Data is contained within the article.
